# The Significance of Short Latency in Mesothelioma for Attribution of Causation: Report of a Case with Predisposing Germline Mutations and Review of the Literature

**DOI:** 10.3390/ijerph182413310

**Published:** 2021-12-17

**Authors:** Sonja Klebe, Ashleigh J. Hocking, Matthew Soeberg, James Leigh

**Affiliations:** 1 Department of Anatomical Pathology, SA Pathology at Flinders Medical Centre, Adelaide, SA 5042, Australia; 2 Department of Anatomical Pathology, Flinders University, Adelaide, SA 5042, Australia; ash.hocking@flinders.edu.au; 3 Asbestos Diseases Research Institute, Concord, NSW 2139, Australia; matthew.soeberg@adri.org.au (M.S.); jleigh@bigpond.com (J.L.)

**Keywords:** mesothelioma, latency, genetic predisposition syndrome, mesothelioma in situ, BAP1, RAD51, TP53

## Abstract

Malignant mesothelioma is a tumour of the serosal membranes, related to asbestos exposure. Median latency is in the order of 40 years in various registries, but small numbers of cases with shorter latencies have long been reported and often dismissed as unrelated to asbestos exposure. However, emerging data regarding the significance of inherited mutations leading to a predisposition to mesothelioma suggest that the causative effect of asbestos may be associated with shorter latencies in a subset of patients. Here, we describe a male patient with germline mutations in RAD51 and p53 who developed peritoneal mesothelioma 8.5 years after well-documented asbestos exposure and discuss the current literature on the subject. Mesothelioma in situ is now a WHO-accepted diagnosis, but preliminary data reveal a potential lead time of 5 or more years to invasive disease, and this is also a factor which may affect the recording of latency (and potentially survival) in the future.

## 1. Introduction

Malignant mesothelioma is a tumour of the serosal membranes, including the pleura, peritoneum, tunica vaginalis testis, and pericardium. Asbestos is the most commonly identified causative agent. No lower threshold level of exposure to asbestos has been delineated below which there is no increase in the risk of mesothelioma, i.e., there is no ‘safe’ level of exposure. Most authorities approach the causation of mesothelioma by asbestos from the perspective of a no-threshold model [1,2]. From the Peto model and its modifications [3,4,5,6], the ‘risk’ and causation of mesothelioma have been related to cumulative asbestos exposure (assessed from intensity, frequency, and duration of exposure) multiplied by time in years raised to the cubic, 3.5th or 4th power, so that other factors being equal, the time elapsed following commencement of exposure is a major determinant of ‘risk’. Epidemiological studies suggest that, for most people, there is no increase in the ‘risk’ (and occurrence) of mesothelioma for at least 10 years following the commencement of exposure, and The Helsinki Criteria [7] adopt a minimum a 10-year latency interval in order to assign the causation of mesothelioma to asbestos. Cases with shorter latency have long been reported [8,9,10,11,12,13,14,15] and are often challenged [16,17], but the reasons for shorter latency were poorly understood. It was usually suggested that there may have been earlier asbestos exposure which had not been recognised, and whilst that may be true for some cases, recent molecular findings suggest otherwise in at least some patients. In addition, mesothelioma is diagnosed earlier, including on cytology and in situ, which may affect latency [18,19]. This may have important consequences for attribution of causation in an individual patient. Here, we discuss a case of short (8.5 years) latency in a patient with well-defined exposure and genetic predisposition which would have increased susceptibility to asbestos [20,21,22,23,24]. We suggest that latencies of less than 10 years may indeed be causative in the development of mesothelioma in some patients, and detailed germline investigation may be warranted in such patients to investigate the possibility of underlying genetic predisposition.

## 2. Materials and Methods

### 2.1. Personal and Family History, Exposure History, and Diagnosis

The clinical presentation, family history, and occupational exposure history were obtained from clinical notes and the patient’s affidavit. The histopathology diagnosis was independently validated.

### 2.2. Genetic Analysis

Whole-genome sequencing was performed on DNA extracted from fresh frozen tissue and matched whole blood. Libraries were prepared using the Illumina TruSeq Nano library method using 200 ng of DNA. Indexed libraries were pooled and sequenced to a depth of 50× for the normal and 100× for tumour using paired 150 bp reads on the Illumina Novaseq 6000 platform. Sequence reads were aligned to build 38 of the genome human reference genome using Burrows–Wheeler Aligner (BWA)-MEM. Variants were detected by at least 2 of the following mutation callers (Mutect2, Strelka2, and Vardict) using the BCBIO pipeline (https://github.com/chapmanb/bcbio-nextgen) (accessed on 26 July 2021). All variants were annotated using the personalised cancer genome reporter (https://github.com/sigven/pcgr) (accessed on 26 July 2021). SNV/InDels in the report were classified according to a five-tiered structure, similar to proposed recommendations [25] also adopting the MLVD framework for the description of clinically relevant cancer variants. Whole-genome sequencing was performed at 100× sequencing depth. Details regarding annotation are provided in Appendix A.

## 3. Results

### 3.1. Personal and Family History, and Exposure History

The patient grew up in Europe with no exposure to asbestos. There was no family history of malignancy. After leaving school, he worked in various employments, as a pastry cook and baker, and as a driver for various companies, with no exposure to asbestos. Aged 55, he obtained work as a truck driver for a demolition company that carried out asbestos removal and demolition work and remained in that position for about 3 to 4 months. He carted building materials to the tip and assisted in demolition work, which included removal of materials containing asbestos, breaking up asbestos–cement sheets by hand, and loading the sheets and debris into the truck, with no protective equipment. There was a personal history of low-grade neuroendocrine tumour of the small bowel (‘carcinoid’) at age 57 which was completely resected, with no chemotherapy and no recurrence at the time of presentation with mesothelioma. There was about a one-month delay between developing abdominal symptoms and the biopsy diagnosis of peritoneal mesothelioma.

### 3.2. Diagnosis

The patient was diagnosed with peritoneal mesothelioma of epithelioid type, aged 63, 8.5 years after the documented exposure to asbestos. There were malignant ascites and peritoneal nodules at laparoscopy which corresponded to infiltrative epithelioid mesothelioma, characterised by positive labelling for mesothelial-related markers calretinin, WT1, CK5.6, and CK7, whilst there was no labelling for carcinoma-related markers TTF1, BerEP4, and CDX2, as well as no labelling for CK20, CD138, neuroendocrine markers chromogranin, and synaptophysin. There was a loss of nuclear labelling for BRCA1-Associated Protein 1 (BAP1).

### 3.3. Genetic Analysis

Germline sequencing demonstrated a pathogenetic mutation in RAD51D, which results in a truncated protein, and a germline TP53 missense mutation. With regard to the tumour, this contained a pathogenic somatic neurofibromatosis 2 (NF2) frameshift mutation and pathogenic loss of function somatic BAP1 mutation, which is in line with the immunohistochemistry result showing loss of nuclear labelling for BAP1.

## 4. Discussion

The development of malignant mesothelioma is dependent upon the following factors in particular:

Toxicology: (i) The inhaled ‘dose’ of asbestos fibres, by way of a no-threshold dose–response relationship—as cumulative asbestos exposure increases, so does the probability and frequency of occurrence of mesothelioma as a consequence;

(ii) Asbestos fibre types: The amphibole forms of asbestos are substantially more potent for mesothelioma induction than white asbestos (chrysotile) on a fibre-for-fibre basis;

Epidemiology: Time elapsed following exposure (e.g., time in years^3.5^).

The Peto model and its modifications [3,4,5] show that the time following commencement of exposure is a major determinant of ‘risk’, i.e., early exposures are more significant for mesothelioma causation than later exposures, with other factors remaining constant. All cumulative exposure adds incrementally to the risk.

The germline RAD51 and p53 mutations in this patient would have led to an acceleration of the biological processes leading to mesothelioma. When asbestos fibres are inhaled, a proportion will be deposited in the lung tissue, and some fibres translocate to the pleura. Asbestos fibres cause genetic damage, including chromosomal alterations. Damage may be caused directly or indirectly. Indirect damage can be mediated by highly reactive free radicals, such as reactive oxygen species (ROS) and reactive nitrogen species (RNS), are generated from the surface of asbestos fibres or from their interactions with macrophages. ROS and RNS have the capacity to damage DNA. [26]. Normal cells have the ability to either repair DNA damage or, in cases in which the DNA is damaged beyond repair, self-destroy by apoptosis [26]. The deposition and persistence of asbestos fibres in conjunction with iron-rich macromolecular aggregates (so-called asbestos bodies) cause chronic inflammation [27] and oxidative stress via ROS, which may lead to DNA damage. Asbestos-activated macrophages release further ROS during unsuccessful attempts of phagocytosis of asbestos fibres, and DNA damage may also occur indirectly via the formation of 8-hydroxy-2′- deoxyguanosine (8-OHdG) adducts [28], with raised blood levels being detectable for years in persons exposed to asbestos [29]. If the cellular damage is not detected or repaired, this results in (somatic) gene mutations, and multiple genetic events are required to cause mesothelioma. Asbestos exposure can induce numerous genes, including a prominent Tp53 response which induces apoptosis, reducing the number of cells that can proliferate [30].

The significance of germline mutations as factors influencing the susceptibility to asbestos and mesothelioma development is increasingly being recognised [20,22,31,32]. In this patient, there was a demonstrated germline mutation in RAD51D. RAD51D mutations are common somatic mutations in mesothelioma [33], and somatic RAD51D mutation is an inclusion criterion in phase 2 clinical trial for mesothelioma and is believed to predict poly ADP ribose polymerase (PARP) inhibitor sensitivity (https://ichgcp.net/clinical-trials-registry/NCT04171700) (accessed on 24 August 2021). RAD51D is needed to produce a protein involved in DNA repair, including the repair pathway of double-stranded DNA breaks which are induced by DNA-damaging agents (such as asbestos) [34,35].

RAD51D is one of the most common cancer susceptibility genes mutated in the germline of patients with solid cancer [36] and is known to predispose to ovarian, breast, and prostate carcinomas (https://www.mycancergenome.org/content/gene/rad51d/) (accessed on 24 August 2021). RAD51D interacts with the BLM gene, which encodes a helicase enzyme that resolves DNA replication forks that have stalled due to DNA damage. Germline BLM deletions have convincingly been shown to increase the susceptibility to asbestos and mesothelioma in clinical, in in vitro and animal studies [37]. Part of the RAD51 pathway is implicated in acting on the BRCA1–BRCA2-dependent repair pathway which includes a possible functional correlation between the two proteins. This patient has a germline mutation in RAD51, likely impacting BRCA-mediated DNA repair, and a somatic mutation in BAP1. Somatic BAP1 deletions are recognised as early events in mesothelioma development [38], and the combination of germline and somatic mutation in this pathway would have likely impacted downstream cell cycle regulation and DNA repair [39,40].

**Figure 1:**
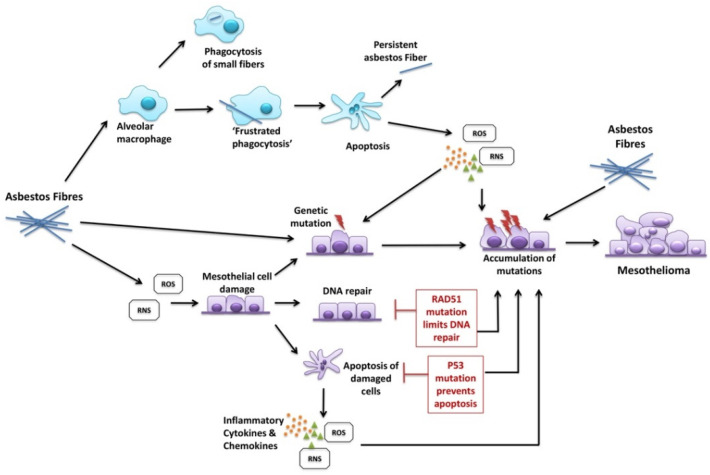
Chronic inflammation after asbestos inhalation causes DNA damage, leading to mutations and the proliferation of malignant cells. Apoptosis acts as a protective mechanism, removing damaged—and possibly malignant—cells. The genetic compromise in DNA repair and reduced effectiveness of the protective apoptotic mechanisms that normally safeguard the cell from malignant transformation would increase the speed at which mutations accumulated.

Asbestos fibres can also interact directly with cells and cause genetic changes, so both the fibres and the secondary chemical messengers in the form of free radicals are implicated at multiple stages in mutagenesis. DNA damage is the critical change that ultimately resulted in the development of mesothelioma, and at each of these incidents, DNA repair would have been impaired as a result of the RAD51 and TP53 germline mutations. TP53 is a tumour suppressor, often referred to as ‘the guardian of the genome’. Its primary function is to induce cell-cycle arrest and apoptosis in response to irreversible DNA damage. Many studies have demonstrated that intact p53 cell-cycle regulation and apoptosis functions are important for preventing tumour development. In addition, p53 has regulatory functions in antioxidant response and DNA repair—both relevant to carcinogenesis as a result of exposure to asbestos [26,41,42,43] (Figure 1). Recently, in a study of genetic changes in mesothelioma, 11 molecular aberrations were found in 6 patients, and 2 mutations were identified in both NF2 and TP53 genes [44]. This study did not distinguish germline from somatic mutations but supports the importance of TP53 mutation in mesothelioma. The high frequency and likely significance of TP53 germline mutations in increasing sensitivity to asbestos and ultimately contributing to mesothelioma development have been observed [44,45].

Continued, repeated DNA damage is the essential pathway to tumour development in mesothelioma, and a defect in the DNA repair gene or apoptosis is likely to accelerate this. There are several genes involved in DNA repair which are associated with a particular pattern, and not all types of DNA repair mechanism are believed to be relevant in mesothelioma development [46], but 52% of mesotheliomas sequenced were shown to have a DNA repair pathway defect, either as a result of a germline or acquired event [47].

In their study of 198 mesothelioma patients, Panou et al. [47] found that germline mutation frequency in cancer susceptibility genes was highest in patients with peritoneal mesothelioma, little or no known asbestos exposure, a second cancer diagnosis, and epithelioid histology. All of those applied to this patient. The authors concluded that minimal-to-no asbestos exposure was the most significant predictor of the presence of a germline cancer susceptibility mutation and noted that further important predictors—younger age and having had a second type of cancer—were not surprising given the known association of cancer susceptibility gene mutations and earlier onset mesothelioma as well as multiple cancers. Based on the higher overall proportion of germline mutations in peritoneal mesothelioma patients, it was suggested that inherited susceptibility may play a larger role in peritoneal mesothelioma. Interestingly, there is overlap in site and cisplatin sensitivity of peritoneal mesothelioma and ovarian cancer, a type of cancer for which 18% to 24% of patients will carry a germline mutation in the RAD51D gene [47].

Increased susceptibility to asbestos toxicity and a faster pace at accumulating mutations would affect the latency. There are many studies reporting mean or median values of over 40 years [12,48,49,50], but some studies showed large interindividual variability in latency from <10 to >70 years [8,48,50]. This variability has been attributed to difficulties in obtaining precise exposure data, the variable intensity of exposure, and individual characteristics (such as genetic predispositions).

The most widely quoted acceptable latency interval for attribution of causation is 10 years, and this is the latency mentioned in the Helsinki criteria [51]. Shorter latencies have long been reported (summarised in Table 1). Chovil and Stewart reported 26 cases of compensated mesothelioma cases with mainly chrysotile exposure. Latency ranged from 6–44 years. [8]. Case reports include pleural mesothelioma in a 28-year old man 8 years after brief exposure to blue asbestos [14], pleural mesothelioma 8.5 years after bystander exposure [10], and pleural mesothelioma 7.5 years after chrysotile exposure [15]. In Greenberg’s report on the British mesothelioma register, a 3.5-year latency was mentioned, and a 7.5-year latency was described after intermittent chrysotile exposure [52]. The Australian Mesothelioma Surveillance program registered 4 cases of 499 with less than 10-year latency [53], and Driscoll and Leigh noted 2 of 1968 cases with less than 10-year latency [54]. In their pooled analysis of six occupational cohort studies, Reid et al. identified one pleural and one peritoneal mesothelioma in Eternit workers, with 7.6- and 7.2-year latency, respectively [55]. In a retrospective analysis of workers in the German Democratic Republic 6 of 332 cases [13,56] had less than 10 years latency. The median latency for the 132 patients with peritoneal mesothelioma in a cohort of 614 British asbestos was only 8.2 years [9].

A study of 2644 mesothelioma cases with asbestos exposure history collected by the Italian Mesothelioma Register (ReNaM) found a median latency of 44.6 years (6–84 years for males and 9–84 years for females) [12]. Finally, Merlo et al. found a minimum latency of 9 years in shipyard workers in Genova [57], and Lacourt reported a latency range of 7–61 years [58].

**Table 1:** Summary of studies reporting less than 10-year latency. Several of the studies did not specify maximum latency. Abbreviations: nd = no data, * the two recorded cases with <10 years latency (3.5 and 7.5 year) had chrysotile exposure; # 4 cases <10 years; ^ 2 cases <10 years; ^&^ 1.8% cases (6 of 332 cases) <10 years; ^+^ 24 of 614 cases <10 years.

Type of Study	Exposure	Site	Mean/ Median Latency	Latency (Range in Years)	Ref.
Compensation Board	Mostly Chrysotile	nd	26.9	6–44	[8]
Case report	Amphibole	Pleura	nd	8	[14,17]
Case report	Chrysotile	Pleura	nd	7.5	[15]
Registry	Various	nd	nd	3.5–65 *	[52]
Surveillance program	nd	nd	37.4	4–66 ^#^	[53]
Registry	nd	nd	43.9	6–77 ^	[54]
Pooled cohort	Various	Pleura	38.4	7.6	[55]
Pooled cohort	Various	Peritoneal	38.4 years	7.2	[55]
Registry	Various	nd	nd, 65.7% > 30	<10–>50 ^&^	[13,56]
Cohort study	Mixed	nd	22.8 all 8.2 peritoneal 2.9 pleural and peritoneal	<10 ^+^—not specified	[9,59]
Case report	Mixed	Pleura	8.5	8.5	[10]
Registry	Various	Pleura	44.6	6–84	[12]
Cohort study	Mixed	Pleura	42.8	9.3—not specified	[57]
Case–control study	Various	Pleura	47	7–61	[58]

These epidemiology-based observations were of uncertain significance at the time but can now be supported by the fact that a high proportion of peritoneal mesothelioma patients have predisposing germline mutations [47]. Taken together, these studies may support the notion that inherited susceptibility may be more common in patients with peritoneal mesothelioma [21,47].

Is a short latency simply explicable by very high exposures? Whilst there is no doubt that an increased dose of asbestos fibres translates to an increased risk of contracting the disease, some of the studies which examined the latency of malignant mesothelioma in association with the intensity of exposure to asbestos have reported conflicting results. One study found no correlation between latency and the asbestos fibre count in lung tissue in 42 cases of malignant mesothelioma linked to occupational exposure in Norway [60], similar to the findings in the above-mentioned study by Frost et al. [9], but the shortfalls of these studies were highlighted [11,59,61]. In contrast, a study of British dockyard workers that estimated the intensity of asbestos exposure from occupational activity found that 41 heavily exposed workers had a shorter latency than 241 less heavily exposed workers (42.0 versus 49.5 years) [62]. D’Agostini reported the association between latency and year of first exposure, year of diagnosis, and industry sector, with construction workers having a shorter latency, compared with workers in the shipyard industry, but the authors drew no conclusion regarding dose [63]. In the registry-based study by Neumann et al., the mean latency period was 37.8 years, and a trend of a higher asbestos burden of the lung/shorter latency periods was suggested [64]. 

Dragani et al. [65] studied 594 patients with malignant mesothelioma data in relation to asbestos body count and fibres (per gram of wet lung tissue). They found an association with younger age at diagnosis for both measures of asbestos quantification in lung tissue.

The still-valid conclusion reached by the comprehensive 1991 report of the Health Effects Institute report indicated that, indeed, ALL exposures contribute to mesothelioma development, in an additive fashion [6]. In a patient with a genetic predisposition that enhances the carcinogenic effects of asbestos, these effects would be amplified.

## 5. Conclusions

Latencies of less than 10 years between exposure to asbestos and diagnosis with mesothelioma have long been described, but they have often been dismissed. Whilst it is of course always possible that an earlier exposure has not been accounted for, many of these reported cases (including this one) are well documented, and studies into genetic predisposition now provide biological reasons for the relative short latencies in some patients. For such patients, germline genetic assessment may be helpful to determine a possible causative role. This is likely to become even more relevant in the future because changes in diagnostic modalities and resultant earlier diagnosis mean that patients are diagnosed earlier than previously, owing to current molecular techniques. Sometimes, this can be much earlier, for example at the in situ stages; progression of in situ disease to diffuse mesothelioma can reportedly take 5 years or more, but these patients are still recognised as having mesothelioma. This may mean that the accepted latency periods that were established by conventional histology may, in the future, not accurately reflect the situation for patients who will be diagnosed earlier. Booth, in 1985, proved prophetic when he stated that ‘It is dangerous…to conclude that because mesothelioma so often occurs with a long latent period between exposure and tumour emergence that an occasional case with a short period of exposure and short (five year) latency cannot happen…’ [17]. These cases do exist and should be appropriately investigated, and large-scale genetic studies may improve our understanding.

## Appendix A

Identification of hotspot variants at low allele frequency: Small nucleotide variants were reported only if they occurred at a variant allele frequency of 10%, except for variants occurring in mutation hotspot regions. Pathogenic hotspot variants in the coding region of the genome (sourced from CIVIC, OncoKb, and CGI) were analysed using Somatic Alterations in Genomev (SAGE) (Priestley et al. 2019). SAGE searched for each hotspot in the tumour BAM files directly and reported evidence of hotspot SNVs and small in-frame indels. Variants in these regions were filtered by variant read base, as well as by mapping quality, exact variant match, allele frequency, tumour allelic depth, and whether detected in the germline. As regards copy number and structural variant analysis, whole-genome analysis of copy number was performed using the PURPLE package, and structural variants were detected using MANTA and BreakPointInspector.
